# Lifestyle of a Roman Imperial community: ethnobotanical evidence from dental calculus of the *Ager Curensis* inhabitants

**DOI:** 10.1186/s13002-019-0334-z

**Published:** 2019-12-04

**Authors:** Alessia D’Agostino, Angelo Gismondi, Gabriele Di Marco, Mauro Lo Castro, Rosaria Olevano, Tiziano Cinti, Donatella Leonardi, Antonella Canini

**Affiliations:** 1 Department of Biology, University of Rome “Tor Vergata”, Via della Ricerca Scientifica 1, 00133 Rome, Italy; 2 Società Cooperativa “Il Bètilo” - Servizi per i Beni Culturali a r. l., Via Remigio De Paolis 15, 00030 San Vito Romano (RM), Italy

**Keywords:** Dental calculus, Cereals, Non-dietary micro-remains, Secondary metabolites, Light microscopy, Gas chromatography mass spectrometry, Passo Corese

## Abstract

**Background:**

The analysis of ancient calcified dental plaque is a powerful archaeobotanical method to elucidate the key role of the plants in human history.

**Methods:**

In this research, by applying both optic microscopy and gas chromatography mass spectrometry on this matrix, a detailed qualitative investigation for reconstructing the lifestyle of a Roman imperial community of the *Ager Curensis* (Sabina Tiberina, Central Italy) was performed.

**Results:**

The detection of animal micro-remains and molecules (e.g., hairs, feather barbules, markers of dairy products), starch granules of several cereals and legumes, pollen (e.g., *Juglans regia* L., *Hedera* sp. L.) and other plant micro-debris (e.g., trichome of *Olea* sp., hemp fibers), and phytochemicals (e.g., Brassicaceae, Lamiaceae herbs, *Ferula* sp., *Trigonella foenum-graecum* L., wine, and *Humulus lupulus* L.) in the dental calculus sample demonstrated that plant-derived foods were regularly consumed together with animal resources.

**Conclusions:**

This nutritional plan, consistent with the information reported in ancient written texts, suggested that the studied population based its own subsistence on both agriculture and husbandry, probably also including beekeeping and hunting activities. All together, these results represent proofs for the comprehension of food habits, phytotherapeutic practices, and cultural traditions of one of the first Roman settlements in the Sabina Tiberina area.

## Background

**Fig. 1:**
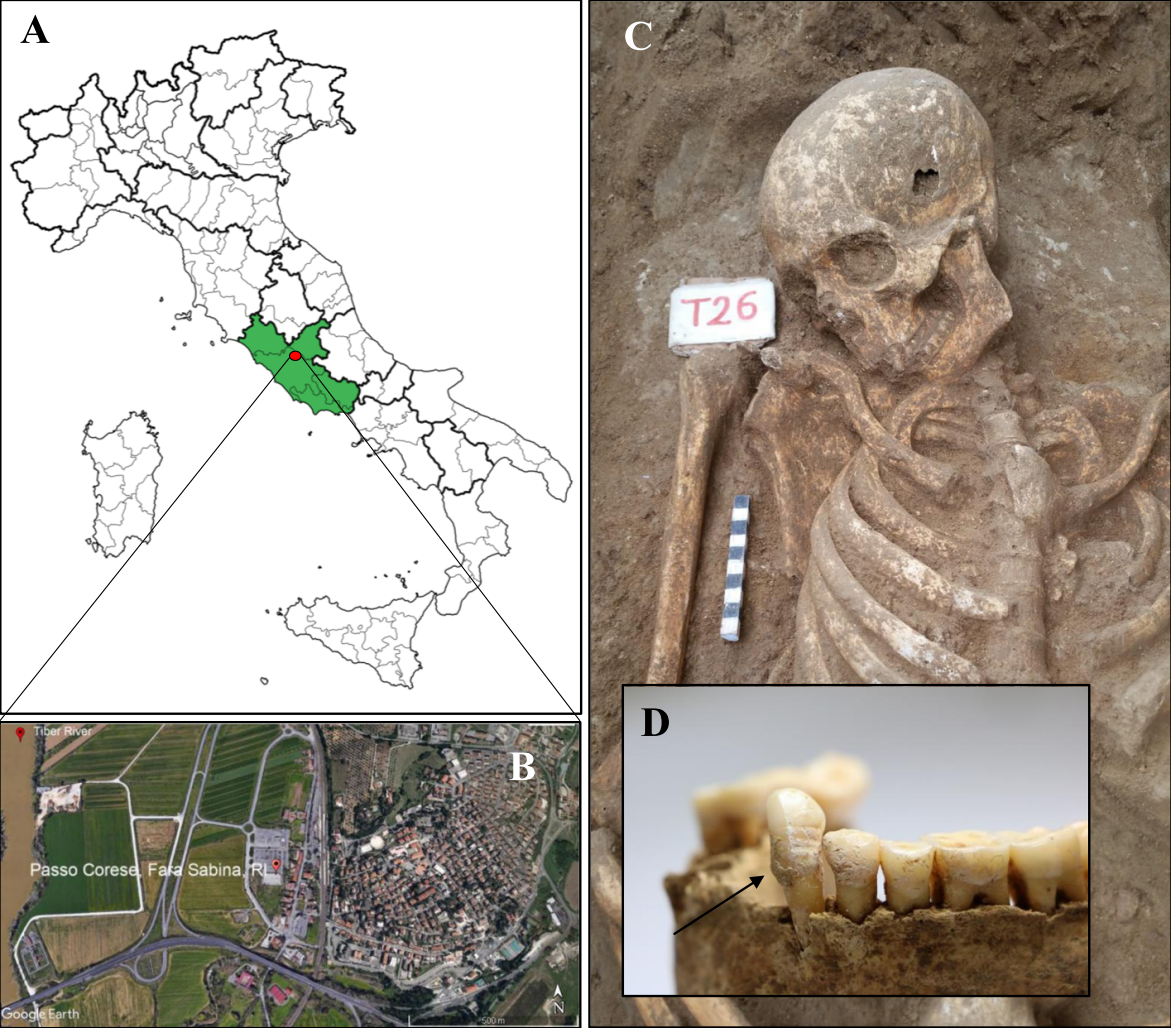
Location of Passo Corese (Fara Sabina, Latium) and inhumation of the burial 26. Map of Italy with the geographical location of the studied site (**a** and **b**, image from Google Earth); representative image of human skeletal remains (**c**) and detail of the relative mandibular dental calculus (**d**)

In 2015, a Roman necropolis has been discovered near the town of Passo Corese (42° 9′ 23.68″ N, 12° 38′ 53.51″ E; Fara in Sabina, Latium, Italy) (Fig. 1a, b). In this archaeological context, 42 tombs of several typologies (e.g., “cappuccina” burials, simple burials) were identified. Radiocarbon dating, performed at the Centre for Diagnostic and Dating (CEDAD, University of Salento) on human bones, has revealed that the burial ground was in use between the first and the third century AD.

Several reports have documented the presence of numerous settlements in the geographic region where the necropolis was unearthed. In that rural territory, *Cures* was considered the main inhabitant center of the *Ager Curensis* (Sabina Tiberina, Central Italy), a hilly area located at about 40 km from Rome [1–3]. As *Via Salaria* and *Tiber* River represented the most important commercial arterial roads of the region, *Cures* gained a hegemonic role at Roman times. The policies of Sulla, Caesar, and Augustus promoted the repopulation of the countryside along the *Tiber* valley, during the following historical periods, favoring the development of rustic villas with the aim to exploit the local agricultural resources [4]. Therefore, the studied necropolis, discovered during excavations of preventive archaeology, could be connected to one of these small villages, whose archaeological remains have not been found yet. The agrarian system at Roman times was very complex, and the selection criteria of the crops were influenced by multiple factors: soil fertility, climate, and ease of storability, processing and yield. Cereals represented the most extensively cultivated crops in Italy. Among them, wheat was the most used, while millet was grown on marginal soils or as summer crop in rotation to other species. In addition, the diet of middle and lower classes also included hemmer, spelt, and barley [5–9]. Beyond the “Mediterranean triad” (grain, olive oil, and wine), the legumes (e.g., peas, chickpeas, lentils, broad beans) were important food sources, especially for Italian rural communities. In particular, they were eaten alone or mixed with wheat or millet [10]. Meat, deriving primarily from poultry, goat, sheep, pork, game, and fish, was mainly associated to festive occasions. In contrast, dairy products and eggs represented the most common animal protein sources, mostly in the sylvo-pastoral economy of hilly regions [5, 6, 10]. Vegetables, like Brassicaceae, onions, and garlic, were complements for poor dishes [5]. A wide variety of fruits and seeds, such as figs, pomegranates, dates, almonds, chestnuts, and walnuts, were abundantly consumed [6, 8, 11]. Wine, beer, and mead (produced by fermentation of grape, malted cereals, and honey, respectively) were the most popular beverages, with also religious and medical value [12, 13]. Beyond food, the ethnobotanical knowledge similarly provided both natural drugs and raw materials, highlighting the key role of the plant kingdom in the ancient world [14–16]. To date, no scientific data testify the economic condition and/or the dietary pattern of the Sabinian communities, including the differences among social classes. However, recent archaeobotanical records contradicted the common idea that some foodstuffs were not available for the lower class, probably due to optimal geographical location, that is access to trades and land fertility [11]. According to all this evidence, the objective of the present research was the investigation of the lifestyle adopted by a Roman community of the *Ager Curensis*. A combined approach between morphological analysis and gas chromatography mass spectrometry was applied on dental plaque, to gain information about the main sources of carbohydrates and the use of edible and/or medicinal plant species by this population. The employment of such type of blended strategy, rarely carried out in literature [17], provided interesting insights on diet and agriculture in Roman central Italy.

## Methods

### Sampling

Archaeological excavations, performed between November and December 2015 and conducted in an area adjacent to the railway station of Passo Corese (Fara in Sabina, Italy), unearthed a Roman necropolis (first–third century AD). Forty-two human skeletons were collected, examined per sex and age at death, and preserved at the “Museo Civico Naturalistico dei Monti Prenestini” (Capranica Prenestina, Rome, Italy). A total of 27 individuals were subjected to dental calculus investigation. Wearing starch-free gloves and using sodium hydroxide (2%) to cleanse tools and work surfaces and reduce contamination, supragingival calculus flakes were removed by an autoclaved metal dental pick from the tooth surface of the inhumates (Fig. 1c, d). In the laboratories of Botany (University of Rome “Tor Vergata,” Italy), specifically reserved to the analysis of ancient biomolecules [18] (where contamination controls were regularly carried out on workspaces, instruments, and supplies), decontamination and sterilization protocols of the mineralized plaque were conducted. In detail, under a sterile vertical laminar flow hood (Heraeus HERAsafe HS12 Type), dental calculus was treated by UV light for 10 min, immersed in 2% sodium hydroxide for 15 min, washed with sterilized water, and dried at 37 °C. To guarantee that environmental contaminants were eliminated, 8 calculus samples were randomly individuated and processed, before and after the cleaning procedures, as follows. Each sample was resuspended in 200 μL of sterilized water, in agitation, for 15 min and directly subjected to light microscopy observation. The results of the control tests (obtained from not decontaminated samples) were reported in Additional file 1. No micro-remain was detected in the samples exposed to decontamination protocols.

### Morphological analysis

To isolate micro-debris from the mineral matrix of the dental plaque (10 mg per sample), after dissolving the calculus in 0.2 M hydrochloric acid for 8 h and performing three washes with sterilized ultrapure water, the pellet was resuspended in 100 μL of bidistilled water and glycerol (1:1), under a sterile hood, and placed on glass slides to be analyzed at optic microscopy (Nikon Eclipse E100). Each micro-remain was observed under white and polarized light, photographed and measured in size by ProgRes CapturePro 2.9.0.1 software. Modern reference image library expressly produced for the identification of animal and plant debris, the laboratory starch experimental collection [19] and literature data were consulted for the taxonomic determination of the ancient pollen and micro-remains [17, 20–24].

### Gas chromatographic mass spectrometry analysis

A qualitative gas chromatographic approach was developed in the present work and applied on ancient dental calculus, as reported. In detail, 10 mg of sample was solubilized in 0.5 mL of 3% hydrochloric acid, overnight. A volume of hexane was added and incubated for 2 h, in agitation. After centrifugation at maximum speed, for 10 min, the hexane fraction was collected, dried out (speed-vac system, Eppendorf AG 22331 Hamburg, Concentration Plus) and derivatized with 60 μL of hexane and 40 μL of the Methyl-8-Reagent (v/v; Thermo Scientific), as reported in the manufacturer’s guidelines. The analysis was performed in a GC-MS QP2010 system (Shimadzu, Japan) equipped with a DB-5 column (Phenomenex; length 30 m × diameter 0.25 mm × thickness 0.25 μm), in triplicate per each sample. The temperature gradient was set as follows: 60 °C for 5 min and then, at a rate of 6 °C/min, the oven reached 150 °C for 5 min, 250 °C for 5 min and 330 °C for 25 min. Helium was employed as carrier gas, at a constant flow of 1 mL/min. The mass spectrum was obtained by electron impact (EI) at 70 eV (scanning from 100 to 700 m/z), ion source and interface temperatures were 230 °C and 320 °C, respectively, and solvent cut time was equal to 6 min. The identification of each molecule was carried out by comparing its mass spectrum with those registered in the NIST Library 14 (similarity values higher than 85%) and on line support [25]. Plant species and food categories ingested by the individuals, at least once during their lifetime, were inferred associating the detected analytes through literature data and scientific food databases [26, 27].

## Results and discussion

Ancient mineralized dental plaque, or dental calculus, is a valuable matrix which is widely employed in archaeological contexts from the 1970s. The plant micro-remains, embedded in this deposit, recorded the variation of the ethnobotanical use of plant species in history [24, 28–34]. The etiology of dental calculus is multifactorial, and individual genetic predisposition, diet pattern, and oral hygiene practices are the most important factors influencing its development. The chemical composition of calculus flakes consists of inorganic salts deriving from saliva, ingested food molecules, and residues of oral microorganisms [35]. Moreover, this ancient deposit can provide additional cultural and environmental information. Indeed, various published works stated the existence of alternative pathways (e.g., accidental or intentional inhalation) for the absorption of dietary and non-dietary debris in tartar [19, 36, 37]. In the present study, dietary customs, phytotherapeutic practices, and other activities besides food of a Roman imperial community of the *Ager Curensis* (first–third century AD) were explored. No significant difference was detected among the samples, according to sex and age at death.

### Morphological analysis

#### Starch granules

**Fig. 2:**
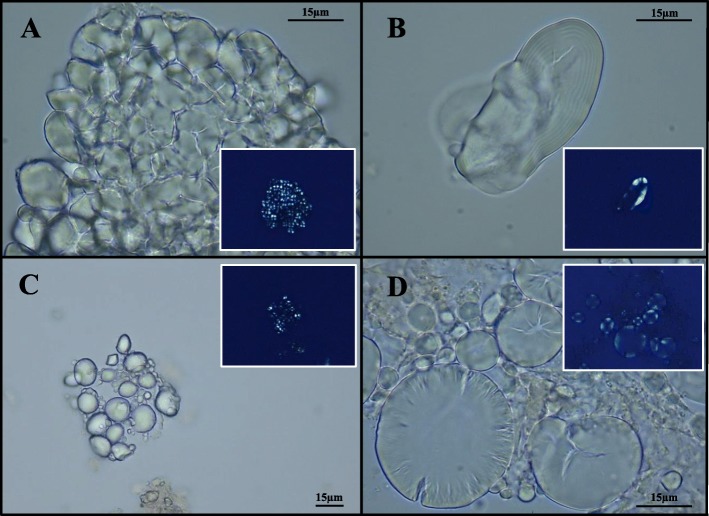
Ancient starch granules at optic microscopy. Representative images of starches found in dental calculus samples: aggregate of *Sorghum* sp. starch grains and relative polarized image (**a**); starch grain of Fabaceae and relative polarized image (**b**); aggregates of Triticeae starch grains and relative polarized images (**c** and **d**)

Optic microscopy revealed the presence of 571 starch grains in all samples (Table 1), some of them shown in Fig. 2. They were clustered into 6 morphotypes and described (Table 2) according to aggregation level (i.e., simple or compound), shape, size, presence of *lamellae* and *hilum*, cracks, and other surface features, and using the international nomenclature code [38] and the starch reference collection hosted in the laboratory of Botany (University of Rome “Tor Vergata,” Italy) [19]. Eleven starches (morphotype I, Table 2) displayed traits consistent with those of legumes (e.g., reniform shape and characteristic longitudinal cleft fissure). In particular, some of them were recognized as *Pisum* sp. L. (e.g., pea) and *Vicia* sp. L. (e.g., broad bean) starch granules, both important sources of proteins for ancient rural communities [39]. On the basis of morphological characteristics (e.g., pyriform shape and size), 11 granules (morphotype II, Table 2) were likely attributable to *Quercus* sp. L. (Fagaceae), typical trees of Mediterranean scrub; the acorns, indeed, were used as foodstuff in famine periods [40]. About these seeds, Pliny the Elder stated in his *Naturalis Historia (16,15)* that they were more palatable if baked. All remaining starches were ascribable to the Poaceae family. Fifty-three polyhedral granules (morphotype III, Table 2), mainly detected in aggregates, were identified as starch sub-units of Poeae caryopses, such as *Avena* sp. L. (e.g., oats). Moreover, 43 ancient starches were assigned to the Paniceae tribe (morphotype IV, Table 2). In detail, these last grains appeared in form of sub-units which showed a faceted surface and radial fissures typical of *Setaria* sp. P. Beauv. (foxtail millet) and *Panicum* sp. L. (millet) starches. Indeed, in Roman times, millet was common ingredient for gruels, while oats also for preparing of malted beers [41, 42]. One hundred and fifty-seven micro-remains (morphotype V, Table 2) corresponded to starches characteristic for Triticeae grasses (i.e., *Hordeum* L. and *Triticum* sp.). These cereals were employed as main components of breads and porridges [42]. In particular, *Triticum dicoccum* L., the emmer wheat, was the most widely cultivated and appreciated crop at that time in Italy [6]. Finally, the last morphotype included 112 starch granules (morphotype VI, Table 2) which displayed morphological and morphometric parameters coherent with those of *Sorghum* sp. Moench. The detection of sorghum suggested import from Africa or direct cultivation of this species in the Roman Empire [41, 43–45]. On the other hand, 184 starches were not taxonomically determined because of lacking diagnostic features. Probably, they were subjected to cooking procedures or enzyme degradation [46].

**Table 1:** Total of starch granules and other micro-remains detected in dental calculus. The amount of each micro-debris identified by microscopic analysis was shown per individual. *Morphotype I*, starch of Fabaceae; *morphotype II*, starch of Fagaceae; *morphotype III*, starch of Poeae; *morphotype IV*, starch of Paniceae; *morphotype V*, starch of Triticeae; *morphotype VI*, starch of *Sorghum* sp.; indeterminate starch granules; O, Oleaceae pollen grain; H, *Hedera* pollen grain; C, *Castanea* pollen grain; J, *Juglans regia* pollen grain; ND, indeterminate pollen grain; F, plant fiber; DH, Cervidae or Bovidae hair; BH, bee hair; T, peltate trichome; GB, fragment of feather barbule of Galliformes; AB, fragments of feather barbule of Anseriformes. In addition, sex (M: male; F: female) and age at death (in years) of each individual were reported

Burial	Sex	Age at death	Morphotype I	Morphotype II	Morphotype III	Morphotype IV	Morphotype V	Morphotype VI	Indeterminate	Total starches per sample	Pollen grains	Other micro-remains
1	M	30–45				1	2		1	4		
2	M	30–45	1			3			1	5		3F
3	M	30–45					3		2	5		
4	F	16–20				1	4		8	13		
6	M	30–45					2		1	3		1F
8	M	30–40	2		16	1	22	92	3	136		3F+1DH
9	M	20–35		8	11		5	1	1	26		
10	M	35–45	1				8	1	2	12	1O	
11	M	13–25	1		5		5	2	20	33		
12	F	24–35				1			2	3		1BH
14	M	20–30				2	5		20	27		
17	F	20–35			6	2	25		12	45		
21	F	35–45					1	3	18	22		
23	M	20–35	1		1		5		16	23		2F
24	F	20–35		1	1			2		4		
25	F	20–35	1				31	1	40	73		
26	M	20–35	2		3		7	2	15	29		
27	M	16–20		2	5	25	10	1	6	49	1H	2 T+1AB
28	M	16–35				2	2	1	2	7	1C+1ND	1GB
29	M	40–45							4	4		
31	M	16–35			5	5	5	2	5	22	1 J+1ND	1AB
34	M	≤ 45	1				5	2		8		
36	F	24–35					2		5	7		
38	M	≤ 45	1				2			3		
39	M	20–35					2	1		3		2F
40	M	25–35					1	1		2		
41	M	25–35					3			3		
Total			11	11	53	43	157	112	184	571	6	18

**Table 2:** Starch morphotypes. Detailed description of the starch morphotypes recovered from the dental calculus of the ancient community

Morphotype	Morphologic and morphometric description	Taxonomic group
I	Irregular ovoid to reniform granules; size range. 20–52 μm in length and 17–38 μm in width; clear concentric *lamellae*; *hilum* is obscured; presence of a longitudinal cleft	Fabaceae
II	Pyriform to reniform granules; size range, 19–35 μm in length and 16–27 μm in width; faintly visible *lamellae*; invisible *hilum*	Fagaceae
III	Multifaceted polyhedral units on one side and dome shaped on the other one; individual granule size, 5–14 μm in length and 5–12 in width; indistinct *hilum* and *lamellae*	Poeae
IV	Polyhedral granules with pentagonal or hexagonal faces and rounded off edges; size range, 3–16 μm in length and 2–15 in width; centric distinct *hilum*; radial fissures; indistinct *lamellae*	Paniceae
V	Discoidal granules; size range, 6–36 μm in length and 4–30 μm in width; indistinct *hilum*; concentric, complete and distinct *lamellae*; sometimes, longitudinal fissures were present.	Triticeae
VI	Ovoidal granules with flattened surfaces; size range, 8–20 μm in length and 7–17 μm in width; indistinct *lamellae*; deep radial fissures starting from a centric *hilum*	*Sorghum* sp.

#### Other plant micro-remains

**Fig. 3:**
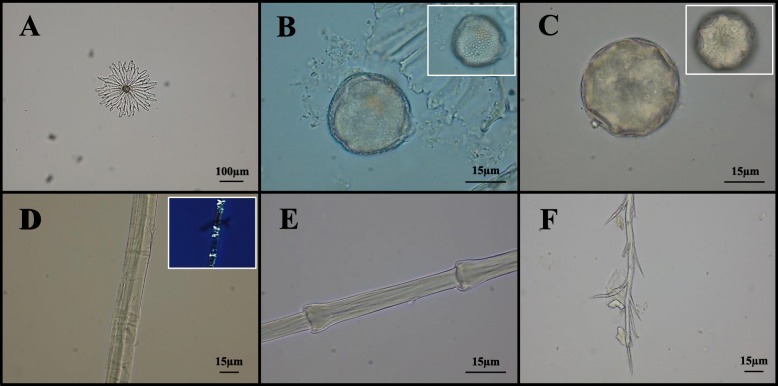
Other micro-remains at light microscopy. Representative images of micro-debris found in dental calculus samples: peltate trichome of *Olea* sp. (**a**); *Hedera* pollen grain (**b**); *Juglans regia* pollen grain (**c**); plant fiber and relative polarized image (**d**); fragment of feather barbule typical of Anseriformes (**e**); bee hair (**f**)

In the samples, a total of six pollen grains were found (Table 1), although two of them were not taxonomically identifiable. The first pollen appeared circular in polar view, medium-sized (35 μm), and polypantoporate (more than 6 apertures with circular germination pores); it was attributed to *Juglans regia* L. (Fig. 3) [47]. The second one was recognized as *Castanea* Mill. pollen grain, according to its aperture condition (tricolporate), shape (prolate), and length (16 μm). Another one displayed some traits consistent with those of *Hedera* pollen; indeed, it was tricolporate, medium-sized (30 μm), characterized by an exine with reticulate ornamentation and a spheroidal and subtriangular shape in polar view (Fig. 3). The last one was isopolar, spheroidal (equatorial diameter, 22 μm), and with reticulate exine; these elements suggested the affinity of this pollen with Oleaceae ones. The pollen grains embedded in the dental calculus, as a consequence of possible accidental aspiration due to breathing, may testify the existence of the relative plant species in the past environments [19, 36]. Indeed, *Juglans regia*, *Castanea sativa* Mill., and *Olea europaea* L. were already diffused in the Mediterranean landscape and highly appreciated by the Romans for their timber and fruits [48, 49]. However, pollen could also be remained entrapped in tartar after ingestion processes. In this regard, it is important to remind that the floral portions of walnut were employed in traditional medicine to treat malaria and rheumatic pains, while chestnut flower extracts as antispasmodic and anti-dysenteric [50, 51]. Last but not least, both honey and other beehive products cannot be excluded as sources of pollen from nectariferous plants, such as chestnut and ivy. Certainly, these matrices were used, in history, not only as sweeteners and food preservatives but also like a powerful natural remedy [52, 53].

In total, 11 plant fibers were recovered from the dental calculus (Table 1, Fig. 3). Under optic microscope, these micro-remains appeared flat, multifibrillar, and not helicoidal, and are characterized by dislocations (or X-nodes) repeated along the strand. Moreover, a Z-twist fibrillar orientation was distinguishable. According to this evidence, the laboratory reference collections for fibers, literature data [54, 55], and both the cultural and chronological context [56–58], all micro-debris could be considered as residues of Cannabaceae plant tissue, most likely hemp. Hemp fibers, obtained by maceration and consequent desiccation of *Cannabis* L. phloem, were used in the Roman period to produce clothes, canvasses, bags, and tough ropes (e.g., for hunting nets) [59–61]. The presence of hemp fibers in the ancient dental calculus might be justified by (i) inhalation during its processing activity [62], (ii) ingestion of food and beverages preserved by hemp sacks [52], and (iii) intake of hemp exudates and extracts for therapeutic purposes. In the second century AD, Galen mentioned the use of sweets containing *Cannabis* to induce hilarity [63]. However, ancient written sources are discordant about the possibility that the Romans knew the medicinal effects of this species [64].

In the dental plaque of one individual, the presence of two non-glandular trichomes of olive (*Olea* sp. L.) leaf were identified (Table 1, Fig. 3). The detection of these superficial multicellular peltate hairs suggested their accidental inhalation or the traditional use of olive leaves as mouth cleanser or in decoction for treating gastro-intestinal diseases, urinary infections, bronchial asthma, and hypertension, a practice widely adopted by the ancient Mediterranean communities [65–68]. However, the most reliable hypothesis seems to be associated with ingestion of olive oil, as Romans used it in almost every dish [8].

#### Animal micro-debris

Among the microfossils, the identification of a branched bee hair appeared curious (Table 1, Fig. 3); it could be associated to consumption of honey or other beehive products [69]. Fragments of feather barbules were found in three individuals of the community (Table 1, Fig. 3). On the basis of its ring-shaped nodes regularly distributed along the structure, one of these micro-debris was attributed to the Galliformes order, which includes, for instance, chickens and pheasants. The other two fragments presented diagnostic triangular-shaped nodes typical of Anatidae family (Anseriformes, e.g., goose, duck) [19, 21, 28, 37]. Another micro-remain showed morphological features ascribable to Cervidae (e.g., *Cervus elaphus* L.) or Bovidae (e.g., *Capra* sp. L.) hair (Table 1). Indeed, this hair showed a multicellular medulla with continuous pattern and structure partially filled lattice, although not well preserved [70]. These last findings were in line with the faunal remains found in many coeval archaeological sites and could have been inhaled during meat preparation (e.g., bird plucking) or derive from fragments of epidermis chewed during the meal. Both wild game and domestic animals, including pigeons, geese, deer, and goats, were exploited by the Romans not only for meat, milk, and eggs, but also for their hides and antlers [6, 71–74].

### Gas chromatography mass spectrometry analysis

GC-MS analysis produced significant results only for 20 individuals. In Additional file 2, the molecules identified in each sample of dental calculus were listed and clustered in biochemical classes. In all cases, replicates always showed similar chromatographic profiles. *N*-alkanes and *n*-alkenes (C_4_ to C_35_) were predominant components of all chromatograms, deriving from decomposition and degradation of oral microbiota or residues of animal or plant edibles [75–77]. These unspecific compounds were not reported in Additional file 2. The detection of methyl esters of saturated fatty acids (e.g., pentadecanoic; *n*-octadecanoic acids), together with unsaturated fatty acids (e.g., 9-octadecenoic; 9,12-octadecadienoic acids), could be considered indicators of consumption of animal fats or plant oils (e.g., oil-rich seeds and fruits, like chestnuts and olives, also supported by previous microscopy evidence) [19, 78]. Polyunsaturated omega-3 fatty acids (e.g., EPA, DHA, and their derivatives), abundant in aquatic sources (e.g., molluscs, algae) and dried fruits (e.g., nuts, hemp seeds) [79], were detected in 9 specimens. Although oil-rich plant foods were commonly employed, the previous evidence could be also justified by consumption of a great variety of fish, or fish sauce (*garum*), as described by several Roman writers [80]. The presence of lactose and 11-octadecenoic acid was indicative of ruminant milk and relative dairy products, widely consumed in the Roman period as alternative protein source, as much as legumes [81, 82]. The identification of terpenes, terpenoids, and their derivatives (e.g., linalool, citronellol, nerolidol, epicubebol, globulol, origanene, menthol, levomenthol, ocimene) suggested the employment of Lamiaceae herbs, typical of Mediterranean area, as food preservatives and flavoring agents [83]. Similarly, other samples revealed the presence of sesquiterpene derivatives ascribable to Apiaceae species (e.g., santalol and shyobunol). In particular, the shyobunol might be considered a potential biomarker of *Ferula* sp. oil [84]. *Ferula assa-foetida* L. (commonly known as asafoetida) was widely used in ancient Rome as a culinary spice or for treating asthma, pneumonia, bronchitis, stomach-ache and flatulence [85]. Isothiocyanic acid, thiocyanic acid, isothiocyanate, isothiocyanatoacetaldehyde dimethyl acetal, and 13-docosenoic acid were also detected by chromatographic analysis; they suggested the intake of cabbages and/or cauliflowers (generally Brassicaceae), which were typical Roman foods [86, 87]. The finding in one sample of tigogenin and smilagenin, two steroidal sapogenins, might indicate both the knowledge and the application of *Trigonella foenum-graecum* L., also called fenugreek [88, 89]. Indeed, Romans used seeds and leaves of this species as sources of food (rich in proteins), spices, drugs (for flatulence, dysentery, and dermatitis), and cosmetics [90, 91]. Dental calculus of one individual presented of a fungal alkaloid, the ergosine, synthesized by *Claviceps purpurea* (Fr.) Tul [92]. As this parasite infests Poaceae species, the previous result suggested a potential contamination of the stored cereals. GC-MS analysis also identified other plant secondary metabolites ascribable to medicinal species, in detail, (i) two azulenes typical of Asteraceae inflorescences, like *Matricaria chamomilla* L. which was used as sedative and anti-inflammatory [14, 93]; (ii) the papaveroline, an isoquinoline alkaloid mainly contained in *Papaver* sp. L. [94], a genus employed in ancient cooking and medicine [95]; (iii) degradation forms of alkaloids (i.e., indole, isoindole, and piperidone) not attributable to specific plant sources; and (iv) digitoxin and cymarin, peculiar markers of *Digitalis* genus and Apocynaceae family, respectively, that were applied in traditional phytotherapy for curing cardiovascular disorders [96, 97]. In particular, digitoxin was detected in the individual of the tomb 23, which was affected by *ante-mortem* compound fracture on both tibia and fibula; this evidence could suggest a potential use of *Digitalis* sp. extracts as powerful energizing drugs [98]. Two calculus samples revealed the presence of phytochemicals (i.e., tartaric acid and pyrogallol) diagnostic of wine [99, 100], an alcoholic beverage with a central role in Roman culture [13, 101, 102]. Indeed, although grape consumption dated back to Neolithic Age [18, 103], wine-making procedures were perfected only in Graeco-Roman era [104]. Usually, wine was diluted in water, aromatized by natural flavoring substances (e.g., lavender, celery, myrtle, rose) or mixed with honey; this last habit might further support the detection of pollen grains in the samples [13]. Finally, the detection of humulol, in one individual, suggested the potential usage of *Humulus lupulus* L., the hop [105]. The inflorescence extract of this species was considered by Romans as a sedative (for insomnia and anxiety), while leaves and shoots were consumed as vegetables in salads [106, 107].

## Conclusions

The archaeobotanical records obtained from dental calculus of the ancient inhabitants of the *Ager Curensis* allowed us outlining the subsistence pattern of this community. This work represents the first scientific study aimed at investigating diet habits and pharmacognosy of a Sabinian population. The detection of plant micro-remains and molecular markers, attributable to different types of cereals and other plant species, indicated a social group whose subsistence was mainly based on agriculture. Grain, such as oat, wheat, and sorghum, represented the main source of carbohydrates, while pulses and acorns provided the protein supply. Evidence of animal consumption also hypothesized husbandry, hunting, and beekeeping activities. Our data documented the putative application of phytotherapeutic remedies, suggesting both traditional knowledge and exploitation of the endemic flora. In particular, plant tissues from Lamiaceae, Apiaceae, Apocynaceae, Papaveraceae, Asteraceae, and Scrophulariaceae species were supposed to be used as raw materials for medicinal formulations. This work revealed the presence of both agricultural and wild species, usable for timber (e.g., chestnut), fiber (i.e., hemp), and food (e.g., olive) production, in the Roman Sabinian landscape. The notable botanical assemblage recorded in this research suggested that the studied community took advantage of multiple and varied natural resources, probably due to their proximity to the Tiber River and Rome. However, supplemental bioarchaeological sampling in the same context might offer further interesting insights about the rural economy of the *Ager Curensis*.

## Supplementary information

**Additional file 1.** Results of contamination control tests on ancient dental calculus before cleaning procedures.

**Additional file 2 **The chemical compounds identified in dental calculus by GC-MS analysis, excluding *n*-alkanes and *n*-alkenes, were reported and classified in biochemical groups, for each sample.

## Data Availability

Available within the paper.

## Abbreviations

GC-MS: Gas chromatography mass spectrometry
EPA: Eicosapentaenoic acid
DHA: Docosahexaenoic acid
