# Supplementary material for: Lifestyle of a Roman Imperial community: ethnobotanical evidence from dental calculus of the Ager Curensis inhabitants
Source: J Ethnobiol Ethnomed. 2019 Dec 4;15:62. doi: 10.1186/s13002-019-0334-z (PMC6894264; doi:10.1186/s13002-019-0334-z)
Supplement: Supplementary file 1 — Additional file 1. Results of contamination control tests on ancient dental calculus before cleaning procedures. [file 13002_2019_334_MOESM1_ESM.pdf]

Supplemental Material 1

Burial	Starch granules	Indeterminate particle	Pollen grains with airsacs	Fungal spores/hyphae	Indeterminate Phytoliths	Calcium oxalate
3		1	2			
8				7	2	
12	1	6				3
17				1		
24	13		1			
25		3		2		1
34		1		4		
40		5			1	
