# Supplementary material for: Lifestyle of a Roman Imperial community: ethnobotanical evidence from dental calculus of the Ager Curensis inhabitants
Source: J Ethnobiol Ethnomed. 2019 Dec 4;15:62. doi: 10.1186/s13002-019-0334-z (PMC6894264; doi:10.1186/s13002-019-0334-z)
Supplement: Supplementary file 2 — Additional file 2 The chemical compounds identified in dental calculus by GC-MS analysis, excluding n-alkanes and n-alkenes, were reported and classified in biochemical groups, for each sample. [file 13002_2019_334_MOESM2_ESM.pdf]

Supplemental Material 2– D’Agostino et al.

Burial 1		
Sugars		Mannose<br>Lactose
Amino acids		Serine
Fatty acids	Saturated	Butanoic acid<br>Pentanoic acid<br>Tridecanoic acid<br>Hexadecanoic acid<br>Heneicosanoic acid<br>Octacosanoic acid<br>Triacontanoic acid
	Unsaturated	11-Octadecenoic acid
Alcohols		1-Decanol<br>6-Tridecanol<br>1-Dodecanol<br>11-Methyldodecanol
Terpens and terpenoids	Monoterpenes and derivatives	Linalool<br>Citronellol, dihydro
	Sesquiterpenes and derivatives	Nerolidol, hexahydro
Phenolic compounds and derivatives		2,6-Dihydroxybenzoic acid<br>1,2-Benzenediol<br>Resorcinol
Other markers		Octanal

Burial 2			
Amino acids			Serine
Fatty acids	Saturated		Hexanoic acid<br>Hexadecanoic acid<br>Tricosanoic acid<br>Pentacosanoic acid<br>Triacontanoic acid
	Unsaturated	omega-3	Docosa-2,6,10,14,18-pentaen-22-al
		omega-6	9,12-Octadecadienoic acid
		omega-7	11-Octadecenoic acid
		omega-9	9-Octadecenoic acid
Alcohols			Tridecan-1-ol<br>1-Dodecanol<br>11-Methyldodecanol
Terpens and terpenoids	Monoterpenes and derivatives		Pinene
	Triterpenoids		Tigogenin<br>Tetrahydrosmilagenin

Burial 3	
Sugars	Galactose

Amino acids			Serine
Fatty acids	Saturated		Hexadecanoic acid<br>Triacontanoic acid
	Unsaturated	omega-3	Docosa-2,6,10,14,18-pentaen-22-al
		omega-9	9-Octadecenoic acid
Alcohols			1-Octanol<br>1-Decanol<br>6-Tridecanol<br>1-Undecanol<br>1-Dodecanol<br>11-Methyldodecanol
Terpens and terpenoids	Monoterpenes and derivatives		Terpinen-4-ol

**Burial 4**			
Amino acids			Serine
Fatty acids	Saturated		Tridecanoic acid<br>Hexadecanoic acid<br>Octadecanoic acid<br>Dodecanoic acid<br>Docosanoic acid<br>Triacontanoic acid
	Unsaturated	omega-3	Doconexent
		omega-7	11-Octadecenoic acid
		omega-12	6-Octadecenoic acid
Alcohols			1-Dodecanol<br>9-Octadecen-1-ol
Terpens and terpenoids	Sesquiterpenes and derivatives		Santalol

**Burial 6**			
Fatty acids	Saturated		Pentanoic acid<br>Tridecanoic acid<br>Tetradecanoic acid<br>Hexadecanoic acid<br>Octadecanoic acid<br>Heneicosanoic acid<br>Docosanoic acid<br>Hexacosanoic acid<br>Octacosanoic acid<br>Triacontanoic acid
	Unsaturated	omega-3	2,6,10,14,18-Pentamethyl-2,6,10,14,18-eicosapentaene<br>5,8,11,14,17-Eicosapentaenoic acid<br>Doconexent
		omega-6	9,12-Octadecadienoic acid
		omega-7	9-Hexadecenoic acid<br>11-Octadecenoic acid
		omega-9	9-Octadecenoic acid<br>13-Docosenoic acid

			15-Tetracosenoic acid
		omega-12	6-Octadecenoic acid
Alcohols			1-Dodecanol<br>3-Dodecanol<br>Tetracosanol-1<br>Octacosanol
Terpens and terpenoids	Monoterpenes and derivatives		1,2-Dihydrolinalool
	Sesquiterpenes and derivatives		Globulol<br>Epicubebol<br>Azulene, 2,3,3a,4,7,8-hexahydro<br>Azulene, 1,2,3,4,5,6,7,8-octahydro
Phenolic compounds and derivatives			Scopoletin
Other markers			Tartaric acid<br>Pyrogallol

**Burial 8**			
Amino acids			Lysine
Fatty acids	Saturated		Butanoic acid<br>Tridecanoic acid<br>Hexadecanoic acid<br>Triacontanoic acid
	Unsaturated	omega-3	Docosa-2,6,10,14,18-pentaen-22-al<br>5,8,11,14-Eicosatetraenoic acid
		omega-7	11-Octadecenoic acid
Alcohols			1-Decanol<br>1-Dodecanol<br>9-Octadecen-1-ol
Terpens and terpenoids	Monoterpenes and derivatives		Citronellol<br>Ocimene
	Sesquiterpenes and derivatives		Shyobunol
Other markers			Vitamin E<br>Isothiocyanic acid<br>2-Decenal<br>7-Hexadecenal<br>15-Heptadecenal

**Burial 9**		
Sugars		Lactose
Amino acids		Serine
Fatty acids	Saturated	Tridecanoic acid<br>Hexadecanoic acid
	Unsaturated	9-Octadecenoic acid
Alcohols		1-Octanol<br>1-Dodecanol<br>11-Methyldodecanol
Terpens and terpenoids	Monoterpenes and derivatives	Levomenthol<br>Ocimene

Burial 10			
Amino acids			Serine
Fatty acids	Saturated		Butanoic acid<br>Tridecanoic acid<br>Hexadecanoic acid<br>Octadecanoic acid<br>Dodecanoic acid<br>Octacosanoic acid
	Unsaturated	omega-7	11-Octadecenoic acid
		omega-9	9-Octadecenoic acid
Alcohols			1-Octanol<br>1-Nonanol<br>1-Decanol<br>1-Dodecanol<br>11-Methyldodecanol<br>11-Tetradecen-1-ol
Terpens and terpenoids	Monoterpenes and derivatives		Citronellol

Burial 11			
Amino acids			Serine
Fatty acids	Saturated		Octadecanoic acid<br>Nonanoic acid<br>Dodecanoic acid<br>Hexadecanoic acid<br>Docosanoic acid<br>Triacontanoic acid
	Unsaturated	omega-3	Docosa-2,6,10,14,18-pentaen-22-al
		omega-7	11-Octadecenoic acid
Alcohols			1-Decanol<br>1-Dodecanol<br>9-Octadecen-1-ol<br>1-Heptacosanol
Terpens and terpenoids	Monoterpenes and derivatives		Menthol<br>Levomenthol<br>Citronellol<br>Pennogenin
Phenolic compounds and derivatives			Phytol

Burial 12		
Amino acids		Serine
Fatty acids	Saturated	Butanoic acid<br>Tridecanoic acid<br>Hexadecanoic acid<br>Octadecanoic acid
	Unsaturated	9,12-Octadecadienoic acid

Alcohols			11-Methyldodecanol<br>1-Decanol<br>1-Dodecanol<br>9-Octadecen-1-ol
Terpens and terpenoids	Monoterpenes and derivatives		Citronellol

**Burial 14**		
Sugars		Lactose<br>Galactitol
Amino acids		Serine
Fatty acids	Saturated	Tridecanoic acid<br>Hexadecanoic acid<br>Octadecanoic acid
	Unsaturated	11-Octadecenoic acid
Alcohols		1-Heptanol<br>1-Dodecanol<br>11-Methyldodecanol<br>Tetracosanol-1
Terpens and terpenoids	Monoterpenes and derivatives	3-Carene
Alkaloids and derivatives		Papaveroline
Other markers		Hexadecanal

**Burial 17**			
Sugars			Inositol
Amino acids			Lysine<br>Serine
Fatty acids	Saturated		Butanoic acid<br>Dodecanoic acid<br>Tridecanoic acid<br>Hexadecanoic acid<br>Heptacosanoic acid<br>Octadecanoic acid<br>Heneicosanoic acid<br>Triacontanoic acid
	Unsaturated	omega-3	Docosa-2,6,10,14,18-pentaen-22-al-
		omega-7	11-Octadecenoic acid
		omega-9	9-Hexadecenoic acid<br>13-Docosenoic acid
Alcohols			1-Heptanol<br>1-Decanol<br>11-Methyldodecanol<br>1-Dodecen-3-ol<br>11-Tetradecen-1-ol
Alkaloids and derivatives			Ergosine
Other markers			Thiocyanic acid<br>Isothiocyanate<br>2-Dodecenal

Burial 21		
Fatty acids	Saturated	Hexadecanoic acid<br>Octadecanoic acid<br>Dodecanoic acid
Alcohols		1-Decanol<br>6-Tridecanol<br>11-Methyldodecanol
Terpens and terpenoids	Monoterpenes and derivatives	3-Carene
Other markers		7-Hexadecenal

Burial 23		
Sugars		Glucopyranose, 1,6-anhydro-
Fatty acids	Saturated	Pentanoic acid<br>Hexadecanoic acid<br>Octadecanoic acid<br>Triacontanoic acid
Alcohols		1-Decanol
Alkaloids and derivatives		Digitoxin

Burial 24		
Fatty acids	Saturated	Dodecanoic acid<br>Hexadecanoic acid<br>Octadecanoic acid
Alcohols		1-Dodecanol<br>11-Methyldodecanol<br>Eicosen-1-ol
Terpens and terpenoids	Sesquiterpenes and derivatives	Humulol
Phenolic compounds and derivatives		Resorcinol
Other markers		2-Decenal

Burial 25			
Sugars			Fucose<br>Sucrose
Amino acids			Serine
Fatty acids	Saturated		Tridecanoic acid<br>Hexadecanoic acid
	Unsaturated	omega-3	Docosa-2,6,10,14,18-pentaen-22-al
		omega-9	9-Octadecenoic acid
Alcohols			1-Decanol<br>1-Dodecanol<br>11-Methyldodecanol<br>1-Tetradecanol<br>9-Octadecen-1-ol

Terpens and terpenoids	Monoterpenes and derivatives		Citronellol
Other markers			Isothiocyanic acid

**Burial 26**			
Fatty acids	Saturated		Hexadecanoic acid
			Heptacosanoic acid
			Octadecanoic acid
			Heneicosanoic acid
			Docosanoic acid
			Triacontanoic acid
	Unsaturated	omega-6	9,12-Octadecadienoic acid
		omega-7	11-Octadecenoic acid
		omega-9	9-Octadecenoic acid
Alcohols			1-Decanol
			11-Methyldodecanol
			Tetracosanol-1

**Burial 27**			
Amino acids			Serine
Fatty acids	Saturated		Pentanoic acid
			Dodecanoic acid
			Hexadecanoic acid
			Docosanoic acid
			Triacontanoic acid
	Unsaturated	omega-3	Docosa-2,6,10,14,18-pentaen-22-al
		omega-9	9-Octadecenoic acid
Alcohols			1-Hexanol
			1-Decanol
			2-Undecen-1-ol
			1-Heptacosanol

**Burial 28**		
Amino acids		Alanine
		Lysine
		Serine
Fatty acids	Saturated	Butanoic acid
		Hexadecanoic acid
		Octadecanoic acid
		Triacontanoic acid
	Unsaturated	9-Octadecenoic acid
Alcohols		1-Hexanol
		1-Heptanol
		1-Octanol
		2-Nonen-1-ol
		1-Dodecanol
		11-Methyldodecanol

<table>
<tr><td colspan="2">Alkaloids and derivatives</td><td>1H-Isoindole-5-carboxylic acid, 2,3-dihydro-1,3-dioxo
1H-Indole
4-Piperidone</td></tr>
<tr><td colspan="2">Other markers</td><td>2-Dodecenal
Decanal
Maltol</td></tr>
</table>

<table>
<tr><th colspan="3">Burial 29</th></tr>
<tr><td rowspan="2">Fatty acids</td><td>Saturated</td><td>Pentanoic acid
Hexadecanoic acid
Octadecanoic acid
Triacontanoic acid</td></tr>
<tr><td>Unsaturated</td><td>7-Hexadecenoic acid</td></tr>
<tr><td colspan="2">Alcohols</td><td>1-Heptanol
1-Nonanol
1-Dodecanol</td></tr>
<tr><td>Terpens and terpenoids</td><td>Monoterpenes and derivatives</td><td>Ocimene</td></tr>
<tr><td colspan="2">Phenolic compounds and derivatives</td><td>Pyrogallol</td></tr>
<tr><td colspan="2">Other markers</td><td>Tartaric acid
2-Dodecenal</td></tr>
</table>

<table>
<tr><th colspan="3">Burial 31</th></tr>
<tr><td colspan="2">Sugars</td><td>Glucopyranose</td></tr>
<tr><td rowspan="2">Fatty acids</td><td>Saturated</td><td>Dodecanoic acid
Hexacosanoic acid
Hexadecanoic acid
Octadecanoic acid</td></tr>
<tr><td>Unsaturated</td><td>9-Octadecenoic acid</td></tr>
<tr><td colspan="2">Alcohols</td><td>1-Dodecanol
11-Methyldodecanol
2-Octadecen-1-ol
11-Tetradecen-1-ol
1-Tricosanol</td></tr>
<tr><td>Terpens and terpenoids</td><td>Monoterpenes and derivatives</td><td>Citronellol</td></tr>
<tr><td colspan="2">Phenolic compounds and derivatives</td><td>1,2-Benzenediol</td></tr>
<tr><td colspan="2">Other markers</td><td>Isothiocyanic acid</td></tr>
</table>

<table>
<tr><th colspan="3">Burial 34</th></tr>
<tr><td colspan="2">Sugars</td><td>Melibiose
Lactose</td></tr>
<tr><td colspan="2">Amino acids</td><td>Serine</td></tr>
<tr><td>Fatty acids</td><td>Saturated</td><td>Dodecanoic acid
Tridecanoic acid
Hexadecanoic acid
Octadecanoic acid
Triacontanoic acid</td></tr>
</table>

<table>
<tr><td></td><td>Unsaturated</td><td>9-Octadecenoic acid</td></tr>
<tr><td colspan="2">Alcohols</td><td>1-Decanol
11-Methyldodecanol
9-Octadecen-1-ol
11-Tetradecen-1-ol</td></tr>
<tr><td>Terpens and terpenoids</td><td>Sesquiterpenes and derivatives</td><td>Sesquithujene</td></tr>
<tr><td colspan="2">Other markers</td><td>Isothiocyanic acid</td></tr>
</table>

<table>
<tr><th colspan="3">Burial 36</th></tr>
<tr><td colspan="2">Amino acids</td><td>Serine</td></tr>
<tr><td rowspan="2">Fatty acids</td><td>Saturated</td><td>Butanoic acid
Hexanoic acid
3-Hydroxydecanoic acid
Hexadecanoic acid
Octadecanoic acid</td></tr>
<tr><td>Unsaturated</td><td>9-Octadecenoic acid</td></tr>
<tr><td colspan="2">Alcohols</td><td>1-Heptanol
1-Decanol
Tridecanol
2-Dodecen-1-ol
1-Hexacosanol
Tetracosanol-1</td></tr>
<tr><td>Terpens and terpenoids</td><td>Monoterpenes and derivatives</td><td>Origanene
Linalool</td></tr>
<tr><td colspan="2">Other markers</td><td>Isothiocyanic acid
Cymarin</td></tr>
</table>

<table>
<tr><th colspan="3">Burial 38</th></tr>
<tr><td>Fatty acids</td><td>Saturated</td><td>Hexadecanoic acid
Octadecanoic acid
Docosanoic acid</td></tr>
<tr><td colspan="2">Alcohols</td><td>1-Pentanol
1-Hexanol
11-Methyldodecanol
1,7-Octanediol</td></tr>
</table>

<table>
<tr><th colspan="4">Burial 39</th></tr>
<tr><td colspan="3">Amino acids</td><td>Serine</td></tr>
<tr><td rowspan="3">Fatty acids</td><td colspan="2">Saturated</td><td>Hexadecanoic acid
Octadecanoic acid</td></tr>
<tr><td rowspan="2">Unsaturated</td><td>omega-3</td><td>Doconexent</td></tr>
<tr><td>omega-6</td><td>9-Octadecenoic acid</td></tr>
<tr><td>Terpens and terpenoids</td><td colspan="2">Monoterpenes and derivatives</td><td>Ocimene
Limonen-6-ol
Citronellol</td></tr>
<tr><td colspan="3">Alcohols</td><td>1-Heptacosanol</td></tr>
</table>

<table>
<tr><td></td><td>1-Dodecanol
1-Triacontanol
Tetracosanol-1</td></tr>
<tr><td>Other markers</td><td>Undecanal</td></tr>
</table>

<table>
<tr><th colspan="3">Burial 40</th></tr>
<tr><td colspan="2">Sugars</td><td>Lactose</td></tr>
<tr><td colspan="2">Amino acids</td><td>Serine</td></tr>
<tr><td rowspan="2">Fatty acids</td><td>Saturated</td><td>Butanoic acid
Heptanoic acid
Hexadecanoic acid
Octadecanoic acid</td></tr>
<tr><td>Unsaturated</td><td>7-Hexadecenoic acid</td></tr>
<tr><td colspan="2">Alcohols</td><td>1-Hexanol
1-Nonanol
Decanol
11-Methyldodecanol</td></tr>
<tr><td>Terpens and terpenoids</td><td>Monoterpenes and derivatives</td><td>Citronellol</td></tr>
<tr><td colspan="2">Other markers</td><td>Thiocyanic acid</td></tr>
</table>

<table>
<tr><th colspan="4">Burial 41</th></tr>
<tr><td colspan="3">Sugars</td><td>Lactose</td></tr>
<tr><td colspan="3">Amino acids</td><td>Serine</td></tr>
<tr><td rowspan="3">Fatty acids</td><td colspan="2">Saturated</td><td>Dodecanoic acid
Hexadecanoic acid
Octadecanoic acid
Cyclopentanetridecanoic acid
Pentacosanoic acid
Triacontanoic acid</td></tr>
<tr><td rowspan="2">Unsaturated</td><td>omega-6</td><td>9,12-Octadecadienoic acid</td></tr>
<tr><td>omega-9</td><td>9-Octadecenoic acid</td></tr>
<tr><td colspan="3">Alcohols</td><td>1-Octanol
1-Decanol
1-Dodecanol
11-Methyldodecanol
11-Tetradecen-1-ol
9-Octadecen-1-ol</td></tr>
<tr><td>Terpens and terpenoids</td><td colspan="2">Monoterpenes and derivatives</td><td>Citronellol
Levomenthol</td></tr>
</table>
